# An Odorant Receptor from the Southern House Mosquito *Culex pipiens quinquefasciatus* Sensitive to Oviposition Attractants

**DOI:** 10.1371/journal.pone.0010090

**Published:** 2010-04-08

**Authors:** Julien Pelletier, David T. Hughes, Charles W. Luetje, Walter S. Leal

**Affiliations:** 1 Department of Entomology, University of California Davis, Davis, California, United States of America; 2 Department of Molecular and Cellular Pharmacology, University of Miami Miller School of Medicine, Miami, Florida, United States of America; INRA - Paris 6 - AgroParisTech, France

## Abstract

**Background:**

Insect odorant receptors (ORs) are heteromers comprised of highly variable odorant-binding subunits associated with one conserved co-receptor. They are potential molecular targets for the development of novel mosquito attractants and repellents. ORs have been identified in the malaria mosquito, *Anopheles gambiae*, and in the yellow fever mosquito, *Aedes aegypti*. However, they are still unknown in the Southern house mosquito, *Culex quinquefasciatus*, which transmits pathogens that cause human diseases throughout the world, including West Nile Virus in the United States.

**Methodology:**

We have employed a combination of bioinformatics, molecular cloning and electrophysiology approaches to identify and characterize the response profile of an OR in *Cx. quinquefasciatus*. First, we have unveiled a large multigenic family of one-hundred-fifty-eight putative ORs in this species, including a subgroup of conserved ORs in three mosquito species. Using the *Xenopus* oocytes expression system, we have determined the response profile of CquiOR2, an antennae-specific OR, which shares high identity with putative orthologs in *Anopheles gambiae* (AgamOR2) and *Aedes aegypti* (AaegOR2).

**Conclusion:**

We show that CquiOR2 is highly sensitive to indole, an oviposition attractant for *Cx. quinquefasciatus*. The response profile of CquiOR2 expressed in *Xenopus* oocytes resembles that of an olfactory receptor neuron housed in the antennal short blunt-tipped sensilla (A2) of *Cx. quinquefasciatus*, which are natural detectors for oviposition attractants. This first *Culex* OR de-orphanized is, therefore, a potential molecular target for screening oviposition attractants.

## Introduction

Insect odorant receptors (ORs), members of a highly divergent multigenic family [1], [2], are expressed in olfactory receptor neurons (ORNs) and housed in olfactory sensilla. Initially, insects ORs were hypothesized to be G-protein coupled receptors (GPCRs), but they have recently been shown to function as heteromeric ligand gated ion channels [3], [4], [5] comprised of at least one copy of a variable odorant-binding OR subunit along with at least one copy of an OR83-like co-receptor [6]. The release of the genome sequences of several species has paved the way for the identification of large families of ORs in different taxa. In mosquitoes, seventy-nine and one-hundred-thirty-one putative OR genes have been identified in *Anopheles gambiae* [7] and *Aedes aegypti* [8], respectively. Recently, a repertoire of fifty ORs from the malaria mosquito *A. gambiae* has been functionally characterized using both the “empty neuron” system of *Drosophila melanogaster* [9] and the *Xenopus* oocyte system [10] providing significant insight into the sense of smell in the malaria mosquito [11]. Interestingly, the authors identified ORs which responded strongly to human derived odorants and may be involved in host recognition.

The complete mapping of olfactory sensilla on the antennae [12], [13] and maxillary palps [14] of the Southern house mosquito *Culex pipiens quinquefasciatus* ( = *Cx. quinquefasciatus*) led to the identification of multiple functional classes of sensilla. Some of these sensilla harbor ORNs highly sensitive to nonanal [13], DEET detectors [15] and ORNs specialized for reception of oviposition attractants [16]. However, the characterization of odorant receptor proteins from the Southern house mosquito remains *terra incognita*. Hitherto, only one OR subunit, the OR83b-like co-receptor CquiOR7 has been characterized at the molecular level [17], but putative odorant-binding subunits are unknown.

In *A. gambiae*, AgamOR2 and AgamOR10 have been shown to respond to a narrow set of chemicals when expressed in heterologous systems [9], [10], [18], [19], including indole and 3-methylindole, which are oviposition attractants for *Culex* mosquitoes [16], [20], [21], [22], [23]. We have mined the genome of *Cx. quinquefasciatus* (The genome sequence of *Culex pipiens quinquefasciatus*; *Culex* Genome Consortium) in attempt to identify ORs likely to be involved in the detection of oviposition attractants. Using bioinformatics approaches we have now identified one-hundred-fifty-eight putative OR genes in the *Cx. quinquefasciatus* genome. Interestingly, two *Culex* genes, *CquiOR2* and *CquiOR10*, are highly related to their putative orthologs in *A. gambiae* (AgamOR2, AgamOR10) and *A. aegypti* (AaegOR2, AaegOR10). We then hypothesized that these ORs are involved in the detection of oviposition attractants in *Cx. quinquefasciatus*. Here, we show that CquiOR2 expressed in *Xenopus* oocytes responds to indole and other oviposition attractants, similar to the response profile of a specific neuron housed in the blunt-tipped trichoid sensilla in *Cx. quinquefasciatus* antennae.

## Results and Discussion

### Identification of putative OR genes in *Cx. quinquefasciatus*

To explore the diversity of the OR family in the genome of *Cx. quinquefasciatus*, we have used the previously identified OR sequences from other dipteran species as probes to look for structurally similar proteins by Blast search [24]. Candidate sequences which displayed significant similarity have been manually screened for characteristic features of the OR family. All resulting proteins exhibited the presence of predicted multiple transmembrane domains, the main hallmark of the OR family. Additionally most candidates, when blasted in NCBI conserved domain database (CDD), exhibited the presence of characteristic motifs (pfam02949, pfam08395) conserved in the insect OR family. Finally, multiple alignments revealed the presence in most candidates of a conserved region near the C-terminus (Ser-Tyr-Ser or Ser-Tyr-Thr) also found in ORs from other dipteran species [8].

Homology searches combined with bioinformatics analysis allowed the identification of one-hundred-fifty-eight putative OR genes including previously identified CquiOR7 [17]. The number of *Culex* OR genes identified here is comparable to the previously characterized OR family from another Culicidae species, *A. aegypti,* which encompasses one-hundred-thirty-one putative OR genes [8]. We have confirmed full-length sequence annotations by cDNA cloning for two genes, *CquiOR2* (XM_001864509/CPIJ014392) and *CquiOR10* (XM_001844036/CPIJ002479) [17]. Accession numbers and structural features of one-hundred-fifty-eight putative OR genes identified in this study, including those originated from VectorBase automated annotations, are described in Table S1.

### Comparative analysis of mosquito ORs

**Figure 1:**
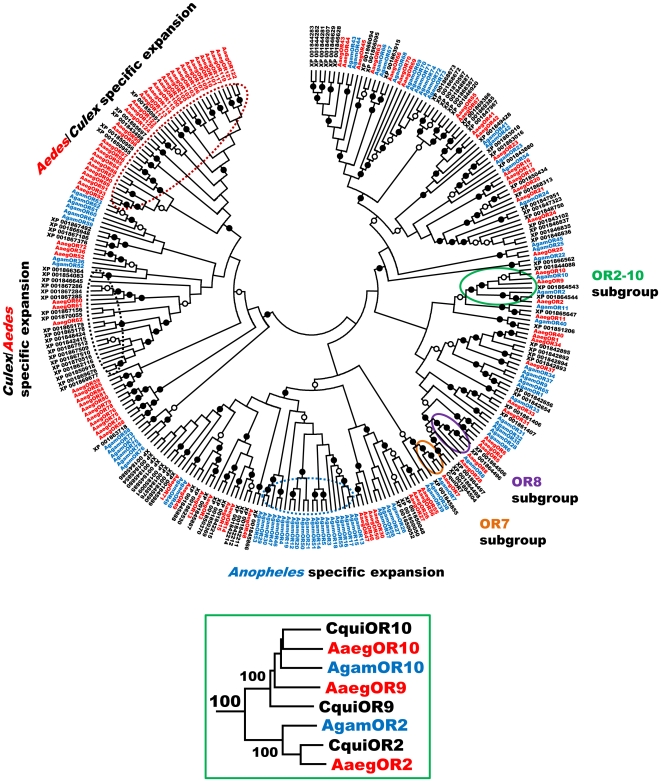
Phylogenetic relationships of mosquito ORs. *Culex* ORs are in black, *Anopheles* ORs are in blue and *Aedes* ORs are in red. Filled circles and empty circles represent 94–100% and 79–93% bootstrap support, respectively. The green box represents relationships in the conserved OR2-OR10 subgroup. CquiOR2 corresponds to (XM_001864509/XP_001864544), CquiOR9 to (XM_001864508/XP_001864543) and CquiOR10 to (XM_001844036/XP_001844088). Major species-specific expansions and conserved OR7 and OR8 subgroups are indicated.

To obtain a better understanding of the relationships among mosquito ORs, we have carried out a phylogenetic analysis using putative amino acid sequences of ORs from three mosquito species, *A. gambiae* [7], [25], *A. aegypti* [8] and *Cx. quinquefasciatus* (this work). A sequence comparison tree reveals the existence of several species-specific lineages as well as different subgroups of conserved ORs within two or three mosquito species (Fig. 1). Focusing in this study on the most conserved OR genes in mosquitoes, we have identified four members of the OR2-OR10 clade in *Cx. quinquefasciatus* which share high identity with related ORs in both *Anopheles* and *Aedes* species (Fig. 1, inset). Three genes (XM_001864507/CPIJ014390; XM_001864508/CPIJ014391; XM_001864509/CPIJ014392) are found at close range on supercontig 3.258, as it has been observed with *AaegOR2*, *AaegOR9* and *AaegOR10* genes, which are also clustered together [8]. These findings suggest that these *Culex* and *Aedes OR* genes might be orthologs. Interestingly, another highly related gene (XM_001844036, CPIJ002479) was found on another genomic location (supercontig 3.32), suggesting a recent *Culex* specific duplication event. Of notice, XM_001864507 and XM_001844036 are both related to OR10 but only the latter was included for phylogenetic analysis as XM_001864507 includes at least three gaps in its predicted sequence.

**Figure 2:**
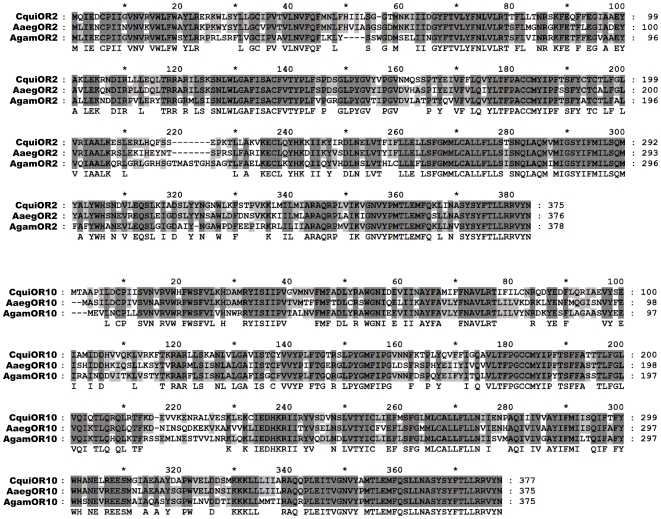
Alignment of mosquito OR2 and OR10 amino acid sequences. Dark grey and light grey shading indicate residues conserved among three species and between two of the three species, respectively.

Among these four genes, XM_001864509 and XM_001844036 displayed the highest identity to *AgamOR2*/*AaegOR2* and *AgamOR10*/*AaegOR10*, respectively, and were, therefore, named *CquiOR2* and *CquiOR10*. Full-length sequences of these two genes were obtained by cDNA cloning and confirmed the predicted annotations, with only minor differences (four amino acid differences in CquiOR2 and a slightly different junction between exons 4 and 5 in CquiOR10). Comparative analysis revealed that, except for OR7 orthologs, OR2 and OR10 are the most conserved within identified ORs in three mosquito species. CquiOR2 shares 81% amino acid identity (91% a. a. similarity) and 70% (83%) with AaegOR2 and AgamOR2, respectively. Likewise, CquiOR10 shares 72% (87%) and 70% (84%) with AaegOR10 and AgamOR10, respectively. Alignment of CquiOR2 and CquiOR10 amino acid sequences with related proteins in the other two mosquito species is shown in Figure 2. Such high sequence conservation for ORs from different species is an interesting feature considering that in general ORs display a high level of divergence [8].

Several other subgroups of related ORs were identified based on sequence similarity and subsequent grouping in the tree (Fig. 1) but only one, the OR8 clade, displayed a similar level of conservation between species. Interestingly, we have identified two putative OR8 orthologs in *Cx. quinquefasciatus* (XM_001864471/CPIJ013954 and XM_001864461/CPIJ013944) which share around 65% and 75% identity with AgamOR8 and AaegOR8, respectively, based on predicted annotations. Recently, functional analysis showed that AaegOR8 acts as an enantioselective detector for (*R*)-(–)-1-octen-3-ol [26] and AgamOR8 was also shown to strongly respond to 1-octen-3-ol [9], [10], [19]. These findings suggest that putative OR8 orthologs in *Cx. quinquefasciatus* may be sensitive to 1-octen-3-ol, a compound which is detected with high sensitivity by sensilla involved in the reception of plant-derived compounds in the maxillary palps of *Cx. quinquefasciatus* [14].

### CquiOR2 and 10 are exclusively expressed in olfactory tissues

**Figure 3:**
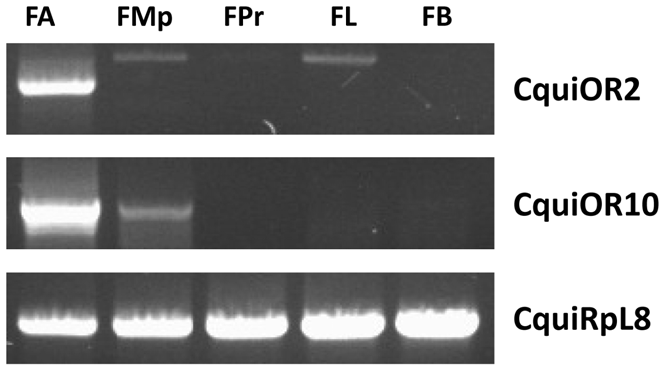
Expression profiles of two ORs in *Cx. quinquefasciatus* female tissues by RT-PCR. Olfactory tissues: antennae (FA); maxillary palps (FMp); proboscis (FPr). Non olfactory tissues: legs (FL); bodies (FB). CquiRpL8 was used as control gene.

Expression patterns of *CquiOR2* and *CquiOR10* have been studied using RT-PCR and cDNA templates prepared from olfactory (antennae, maxillary palps and proboscis) and non-olfactory tissues (legs and bodies) of adult females. *CquiOR2* was detected only in antennae, which is involved in the reception of oviposition attractants [13], [16]. On the other hand, *CquiOR10* was detected in both antennae and maxillary palps, but not in non-olfactory tissues (Fig. 3). Corresponding PCR products were further cloned and sequenced to confirm *CquiOR2* and *CquiOR10* identities. Given that maxillary palps are involved in the reception of plant-volatile compounds and carbon dioxide [14], we reasoned that CquiOR2 is more likely to be involved specifically in the reception of oviposition attractants. Therefore, functional studies were focused on CquiOR2.

### Functional expression of CquiOR2

Previously, AgamOR2 has been shown to strongly respond to indole, 3-methylindole, and 2-methylphenol [9], [10], [18], [19], whereas AgamOR10 has been shown to strongly respond to indole, 3-methylindole and 4-methylphenol [9], [10], [19]. Indoles and cresols have been demonstrated to function as oviposition attractants for *Culex* mosquitoes [16], [20], [21], [22], [23]. Displaying an expression profile restricted to antennae, CquiOR2 was selected for functional characterization in *Xenopus* oocytes to decipher its ligand specificity. Full-length coding sequence of CquiOR2, as well as CquiOR7 [17], the necessary OR83b-like co-receptor, were cloned into pGEMHE [27] for *in vitro* expression in *Xenopus laevis* oocytes.

**Figure 4:**
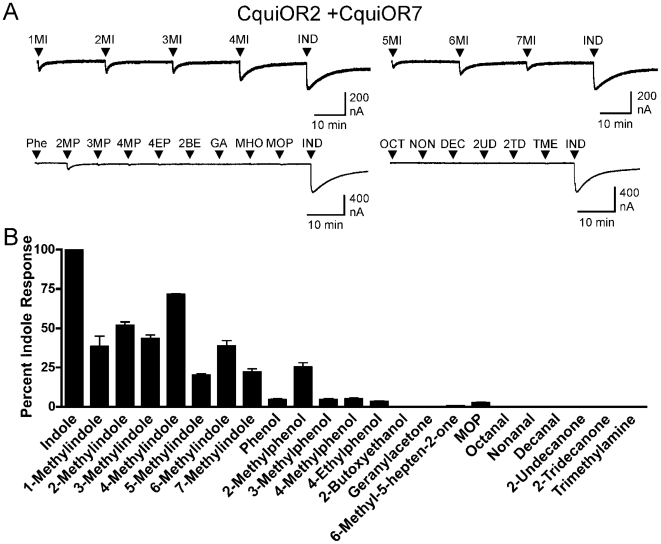
CquiOR2 + CquiOR7 responds to indole, various methylindoles and 2-methylphenol. *Xenopus* oocytes expressing CquiOR2 + CquiOR7 were challenged with a panel of odorant compounds, each applied for 20 s at 10 µM. **A**) *Upper left trace*, an oocyte expressing CquiOR2 + CquiOR7 is challenged with 1-methylindole (1MI), 2-methylindole (2MI), 3-methylindole (3MI), 4-methylindole (4MI) and indole (IND). *Upper right trace*, an oocyte expressing CquiOR2 + CquiOR7 is challenged with 5-methylindole (5MI), 6-methylindole (6MI), 7-methylindole (7MI) and indole (IND). *Lower left trace*, an oocyte expressing CquiOR2 + CquiOR7 is challenged with phenol (Phe), 2-methylphenol (2MP), 3-methylphenol (3MP), 4-methylphenol (4MP), 4-ethylphenol (4EP), 2-butoxyethanol (2BE), geranylacetone (GA), 6-methyl-5-hepten-2-one (MHO), mosquito oviposition pheromone (MOP) and indole (IND). *Lower right trace*, an oocyte expressing CquiOR2 + CquiOR7 is challenged with octanal (OCT), nonanal (NON), decanal (DEC), 2-undecanone (2UD), 2-tridecanone (2TD), trimethylamine (TME) and indole (IND). **B**) Quantification of current responses of CquiOR2 + CquiOR7 expressing receptors. All responses are normalized to the response of the same oocyte to 10 µM indole (mean ± SEM, n = 3–4).

**Figure 5:**
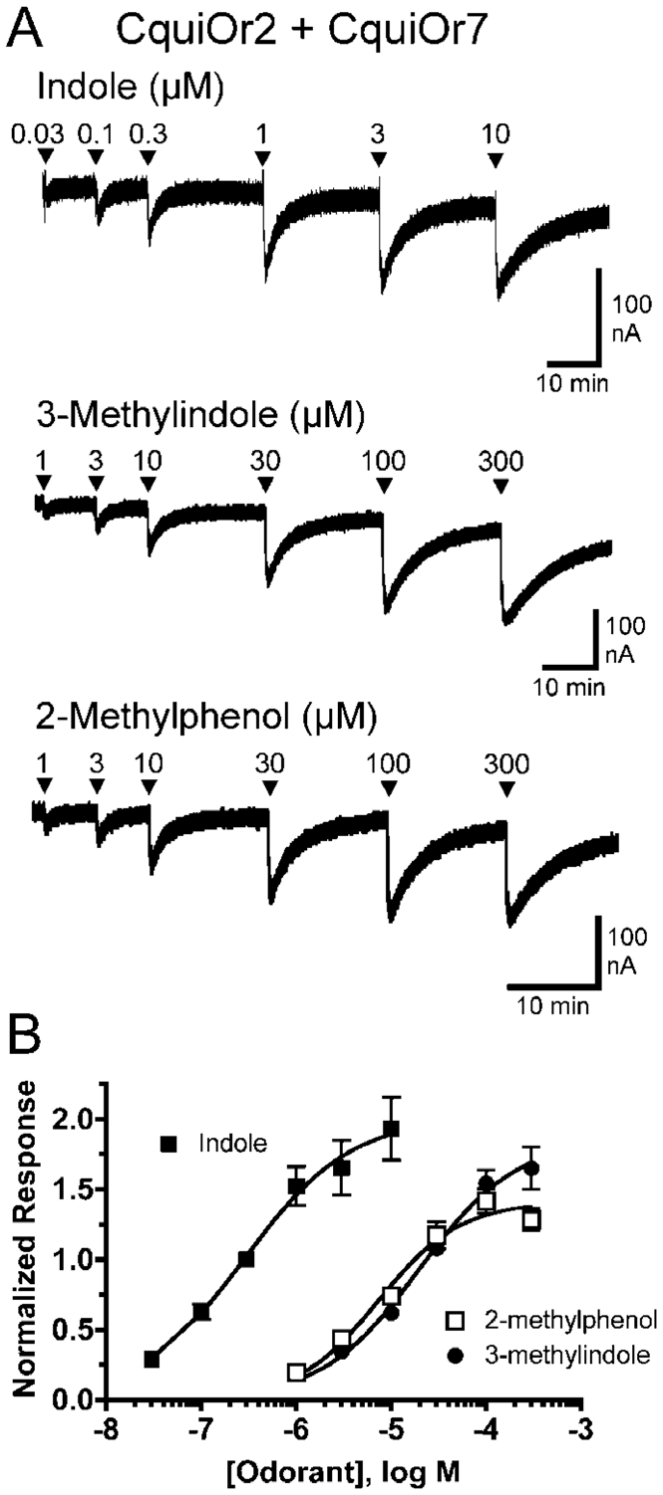
CquiOR2 + CquiOR7 is highly responsive to indole. **A**) *Upper trace*, an oocyte expressing CquiOR2 + CquiOR7 is challenged with 20 s applications of a range of concentrations of indole. *Middle trace*, an oocyte expressing CquiOR2 + CquiOR7 is challenged with 20 s applications of a range of concentrations of 3-methylindole. *Lower trace*, an oocyte expressing CquiOR2 + CquiOR7 is challenged with 20 s applications of a range of concentrations of 2-methylphenol. **B**) Concentration-response relationships for CquiOR2 + CquiOR7 expressing oocytes when activated with a range of indole, 3-methylindole and 2-methylphenol concentrations. All data are normalized to the response of each oocyte to 300 nM indole and the curves were fit as described in Materials and Methods (means ± SEM; n = 5–16). EC_50_ and n_H_ values are provided in Table 1.

Oocytes expressing CquiOR2 + CquiOR7 were screened with a panel of compounds, each applied for 20 s at a concentration of 10 µM (Fig. 4). Indole elicited the largest current responses, but the receptor also responded well to each of the methylindoles and 2-methylphenol. To provide more detail about the sensitivity of the CquiOR2 + CquiOR7 receptor, we performed concentration response analysis for indole and each of the compounds that yielded responses that were at least 20% of the response to indole. Indole was the most potent of the tested compounds, activating the CquiOR2 + CquiOR7 receptor with an EC_50_ of 280 nM (Fig. 5, Table 1). The receptor was also activated by the oviposition attractants 3-methylindole and 2-methylphenol, with EC_50_ values of 20 µM and 7.3 µM, respectively (Fig. 5, Table 1). In addition, each of the other methylindoles were able to activate the receptor with EC_50_'s ranging from 3.0 µM for 6-methylindole to 20 µM for 1-methylindole (Fig. S1, Table 1). Interestingly, while several of the compounds (1-methylindole, 2-methylindole, 3-methylindole) displayed relative efficacies (maximal responses) similar to that of indole, the other compounds (4-methylindole, 5-methylindole, 6-methylindole, 7-methylindole, 2-methylphenol) had lower relative efficacies than indole (Table 1).

**Table 1:** Functional potencies and relative efficacies for activation of CquiOR2 + CquiOR7.

Compound	EC_50_ (µM)	n_H_	Relative Efficacy
Indole	0.28±0.12	0.8±0.2	100
1-Methylindole	20±6	1.3±0.5	84±9
2-Methylindole	7.5±4.1	0.9±0.4	106±16
3-Methylindole	20±6	0.9±0.2	92±8
4-Methylindole	3.6±0.8	1.0±0.2	60±4
5-Methylindole	8.6±2.0	1.1±0.2	59±4
6-Methylindole	3.0±0.6	0.9±0.2	69±4
7-Methylindole	18±3	1.1±0.2	67±4
2-Methylphenol	7.3±1.5	1.0±0.2	70±9

EC_50_ and Hill coefficient (n_H_) values were derived by fitting the data in Figure 5 and Figure S1 to a Hill equation (see Materials and Methods). Relative efficacies are the maximum values derived from fitting the data and are expressed as a percentage of the maximum value for indole. Values are the mean ± SEM derived from concentration-response data from 5–16 separate oocytes.

### with response profiles recorded from *Culex* antennae

Previously, we have shown by direct electrophysiological recordings from *Cx quinquefasciatus* female antennae that indole elicits strong excitatory responses from a type of trichoid sensillum characterized by short length and blunt tip (See Fig. S4, in [13]). This sensillum, classified as type A2 after McIver [28], houses two ORNs as indicated by the spontaneous firing of two distinct amplitudes [13]. The neuron with a larger spike amplitude (ORN-A) was demonstrated to be highly sensitive to nonanal [13], an attractant for host-seeking females [13], which also elicits egg deposition by gravid females [16]. The neuron sensitive to indole, ORN-B, is characterized by spikes with smaller amplitude (See Fig. 4B,C in [13]). Of all the indole-related compounds tested individually, indole elicited the strongest dose-dependent responses (Fig. S4 in [13]). The response threshold was at least three orders of magnitude higher when the same sensilla were challenged with methyl derivatives of indole [13]. The indole-sensitive ORN-B also responded, albeit with lower sensitivity, to phenolic compounds, with 2-methylphenol (*o*-cresol) eliciting the strongest response. The response thresholds for 3- and 4-methylphenol (*m*- and *p*-phenol, respectively) were three orders of magnitude higher than that observed for 2-methylphenol (Fig. S4 in [13]).

Taken together these findings suggest that ORN-B in the blunt-tipped trichoid sensilla A2 in *Cx. quinquefasciatus* female antennae may express CquiOR2–an odorant receptor sensitive to *Culex* oviposition attractants. The electrophysiological responses elicited by stimulating CquiOR2-expressing *Xenopus* oocytes with indole were similar to the profiles obtained by single sensillum recordings from A2 [13]. Both responded to indole in a dose-dependent manner and with high sensitivity (low threshold). Additionally, 2-methylphenol elicited the best responses for phenolic compounds in the mosquito antennae and *Xenopus* oocytes. The natural and heterologous systems did differ in the degree of the selectivity for indole over the methylindoles. CquiOR2 expressed in the *Xenopus* oocyte system is 10- to 70-fold selective for indole over the various methylindoles, while single sensillum recordings from A2 show a three orders of magnitude selectivity for indole [13]. A possible explanation for this discrepancy is that odorant-binding proteins (OBPs) [29] may contribute to and enhance the selectivity of the mosquito olfactory system. Interestingly, we have recently demonstrated that knockdown of an OBP from *Cx. quinquefasciatus*, CquiOBP1, by RNA interference generated a phenotype with reduced electroantennographic (EAG) responses to indole and other oviposition attractants, but no significant changes in responses to nonanal [30]. These experiments with OBP gene silencing and heterologous expression of an OR sensitive to oviposition attractants suggest that OBPs may contribute to both selectivity and sensitivity of insect's olfactory system. Similarly, pheromone-binding proteins (PBPs) have been shown to contribute to the sensitivity [31] and selectivity [32] of moth's reception of sex pheromones. Thus, it is likely that both odorant receptors and odorant-binding proteins contribute to the remarkable selectivity and sensitivity of the insect's olfactory system.

### Comparison with *Anopheles gambiae* ORs

Indole and 3-methylindole, demonstrated here to stimulate CquiOR2, were among the most narrowly tuned odorants in *An. gambiae* ([9], odorants #5 and #1, respectively). Furthermore, AgamOR2 [9], [10] was among the most narrowly tuned ORs, indicating it detects important chemical cues with high specificity. In brief, one of the most narrowly tuned ORs in *An*. *gambiae*, AgamOR2, activates in response to one of the most narrowly tuned odorants, indole. Therefore, it is conceivable that indole plays a significant role in *Anopheles* chemical ecology. *Culex* eggs are deposited in rafts of 200–250 eggs confined to small areas, offering an opportunity for *Culex* population control at this point of their life cycle. Consequently, oviposition behavior has been more thoroughly studied in *Culex* mosquitoes. Indeed, indole and 3-methylindole have been demonstrated in indoor bioassays and field tests to be attractants for gravid *Culex* mosquitoes [16], [20], [21], [22], [23]. An exciting area for future research will be to investigate the response profiles for the related receptor in *Aedes aegypti*, AaegOR2.

### Conclusion

We have identified one-hundred-fifty-eight putative ORs in the genome of *Cx. quinquefasciatus*. Large scale annotations and/or cDNA cloning should now be performed to confirm the functionality of these putative ORs. Here we de-orphanized CquiOR2, which is sensitive to oviposition attractants. These findings open up new avenues for reverse chemical ecology-based approaches [33] aimed at the development of better oviposition attractants by using OBPs [16] as well as ORs as molecular targets.

## Materials and Methods

### Identification of putative OR sequences from *Cx. quinquefasciatus* genome

A predicted peptide sequences database of the whole genome of *Cx. quinquefasciatus* (CpipJ1.2 geneset) available at VectorBase (http://cpipiens.vectorbase.org/index.php) was entered into BioEdit v7.0.9.0 [34] to perform homology searches using Blastp algorithm [24]. Available OR sequences of two mosquito species, *A. gambiae* (seventy-nine sequences) [7] and *A. aegypti* (one-hundred-thirty-one sequences) [8] were used as queries in Blast searches. Candidates were further blasted in NCBI conserved domain database (CDD) to identify motifs conserved of the insect OR family (pfam02949: 7tm Odorant receptor and pfam08395: 7tm Chemosensory receptor). Presence of multiple transmembrane domains was predicted using TMHMM server v2.0 (http://www.cbs.dtu.dk/services/TMHMM/). Multiple alignments and calculation of sequence identities and similarities were made using GeneDoc software (http://www.nrbsc.org/gfx/genedoc/ebinet.htm).

### Phylogenetic analysis of mosquito ORs

Amino acid sequences of putative ORs from three mosquito species were combined to create an entry file for phylogenetic analysis in MEGA 4.0.2 [35]. An unrooted consensus neighbor joining tree was generated based on 1000 bootstrap replicates with pairwise gap deletions. Seventy-nine *A. gambiae* ORs, one-hundred-and-one *A. aegypti* ORs and one-hundred-and-three *Cx. quinquefasciatus* ORs were used in this study. Twenty-one pseudogenes (P) and nine incomplete sequences of *A. aegypti* were omitted (AaegOR12, 18, 22P, 29P, 32P, 35, 38P, 39, 51P, 53, 54P, 57P, 64P, 68P, 73P, 77P, 82P, 83P, 86, 108P, 112P, 116P, 118P, 120P, 126, 127, 128P, 129P, 130, 131P). Fifty-five putative *Cx. quinquefasciatus* ORs were omitted (see Table S1) as they were likely incomplete and/or looked to be partially wrongly annotated sequences when subjected to multiple alignments comparison. Nomenclature of *A. gambiae* and *A. aegypti* ORs used in this study follow the nomenclature established in [7] and [8], respectively.

### Expression patterns and cloning of full-length *CquiOR2* and *CquiOR10*

*Cx. quinquefasciatus* mosquitoes, tissues dissection, RNA extraction and cDNA synthesis were performed as described in [29] with minor modifications. Gene specific primers were designed based on gene annotations to amplify full-length coding sequences of *CquiOR2* (XM_001864509/CPIJ014392) and *CquiOR10* (XM_001844036/CPIJ002479). Antennae, maxillary palps, proboscis, legs and bodies (thorax and abdomen without head/legs) cDNAs from female adult mosquitoes (one-to-seven-day-old) were used as templates for tissue-specificity study. PCR reactions were carried out using equivalent amount of cDNA and one unit of GoTaq DNA polymerase (Promega, Madison, WI) in a final volume of 25 µl. Integrity of each cDNA template was confirmed by the amplification of a ribosomal L8 protein encoding gene (CquiRpL8, XM_001841875). For cloning, full-length sequences of *CquiOR2* and *CquiOR10*, as well as the necessary co-receptor *CquiOR7* (ABB29301) [17], were amplified from female antennal cDNA using Pfu Ultra II polymerase (Stratagene, La Jolla, CA). PCR products were purified using QIAquick Gel Extraction kit (Qiagen, Valencia, CA) and ligated into pBlueScript SK (+) (Stratagene). Ligation products were used to transform One Shot OmniMAX competent cells (Invitrogen, Carlsbad, CA) and positive clones were grown in LB medium containing ampicilline. Plasmids were purified using QIAprep Spin Miniprep kit (Qiagen) and sent for sequencing (Davis Sequencing Inc, Davis, CA). *CquiOR2* and *CquiOR10* cDNA sequences were deposited into GenBank. Accession numbers are GU945396 and GU945397 for CquiOR2 and CquiOR10, respectively.

flCquiOR2up: 5′-ATGCAGATCGAAGACTGCCCCAT-3′

flCquiOR2do: 5′-TTAGTTGTAAACACGACGCAGCA-3′

flCquiOR10up: 5′-ATGACCGCGGCACCCATTTTGGACT-3′

flCquiOR10do: 5′-TCAATTATAAACGCGTCTCAGCAGGG-3′

flCquiOR7up: 5′-ATGAACGTCCAGCCGACCAAGTAC-3′

flCquiOR7do: 5′-TTACTTCAGCTGCACCAACACCAT-3′

CquiRpL8: 5′-AGTCGTGAAGCACATCATCCACG-3′

CquiRpL8: 5′-GCCTTACCGATGTGCTGATGGTT-3

### Expression of ORs in *Xenopus* Oocytes

Oocytes were surgically removed from mature *Xenopus laevis* frogs (Nasco). The care and use of *Xenopus laevis* frogs in this study were approved by the University of Miami Animal Research Committee and meet the guidelines of the National Institutes of Health. Follicle cells were removed by treatment with Collagenase B (Boehringer Mannhem) for 2 h at room temperature. *CquiOR2* and *CquiOR7* were transferred into pGEMHE [27]. Capped cRNA encoding each OR subunit was generated using mMessage mMachine kits (Ambion). 25 ng of cRNA encoding each OR subunit was injected into Stage V-VI *Xenopus* oocytes. Oocytes were incubated at 18°C in Barth's saline (in mM: 88 NaCl, 1 KCl, 2.4 NaHCO_3_, 0.3 CaNO_3_, 0.41 CaCl_2_, 0.82 MgSO_4_, 15 HEPES, pH 7.6, and 100 µg/ml amikacin) for 2–5 days prior to electrophysiological recording.

### Electrophysiology and Data Analysis

Odorant-induced currents were recorded under two-electrode voltage clamp from oocytes expressing ORs, using an automated parallel electrophysiology system (OpusXpress 6000A; Molecular Devices). Oocytes were perfused with ND96 (in mM: 96 NaCl, 2 KCl, 1 CaCl_2_, 1 MgCl_2_, 5 HEPES, pH 7.5). Odorants were diluted in ND96 and applied for 20 s at a flow rate of 1.65 ml/min with extensive washing in ND96 (7–20 min at 4.6 ml/min) between applications. Current responses approached a plateau during the 20 sec application (Figure S2). Micropipettes were filled with 3 M KCl and had resistances of 0.2–2.0 MΩ. The holding potential was −70 mV. Current responses were filtered (4-pole, Bessel, low pass) at 20 Hz (-3 db), sampled at 100 Hz and were captured and stored using OpusXpress 1.1 software (Molecular Devices). Initial analysis of electrophysiological data was done using Clampfit 9.1 software (Molecular Devices). Curve fitting of concentration-response data was done using Prism 4 (Graphpad). Concentration-response data were fit to the equation: I = I_max_/(1+(EC_50_/X)^n^) where I represents the current response at a given concentration of odorant, X; I_max_ is the maximal response; EC_50_ is the concentration of odorant yielding a half maximal response; n is the apparent Hill coefficient.

## Supporting Information

Figure S1 Concentration-response analysis for methylindoles. The concentration-response relationships for CquiOR2 + CquiOR7 expressing oocytes when activated with a range of methylindole concentrations are shown. All data are normalized to the response of each oocyte to 300 nM indole and the curves were fit as described in Materials and Methods (means ± sem; n = 6–9). The data for indole is from Figure 5 and is shown for comparison. EC_50_ and nH values are provided in Table 1.
(1.60 MB TIF)

Figure S2 Kinetics of the response of CquiOR2 + CquiOR7 to 10 µM indole. An oocyte expressing CquiOR2 + CquiOR7 is challenged with a 20 s application of 10 µM indole (IND). Note that the response to indole approaches a plateau during the 20 second application. The response diminishes very slowly during washout, suggesting that the receptor is supersaturated and that indole is likely to be highly potent. This is borne out by the concentration-response data in Figure 5.
(1.07 MB TIF)

Table S1 (0.22 MB DOC)
