# Supplementary figures and images for: An Odorant Receptor from the Southern House Mosquito Culex pipiens quinquefasciatus Sensitive to Oviposition Attractants
Source: PLoS One. 2010 Apr 8;5(4):e10090. doi: 10.1371/journal.pone.0010090 (PMC2851645; doi:10.1371/journal.pone.0010090)

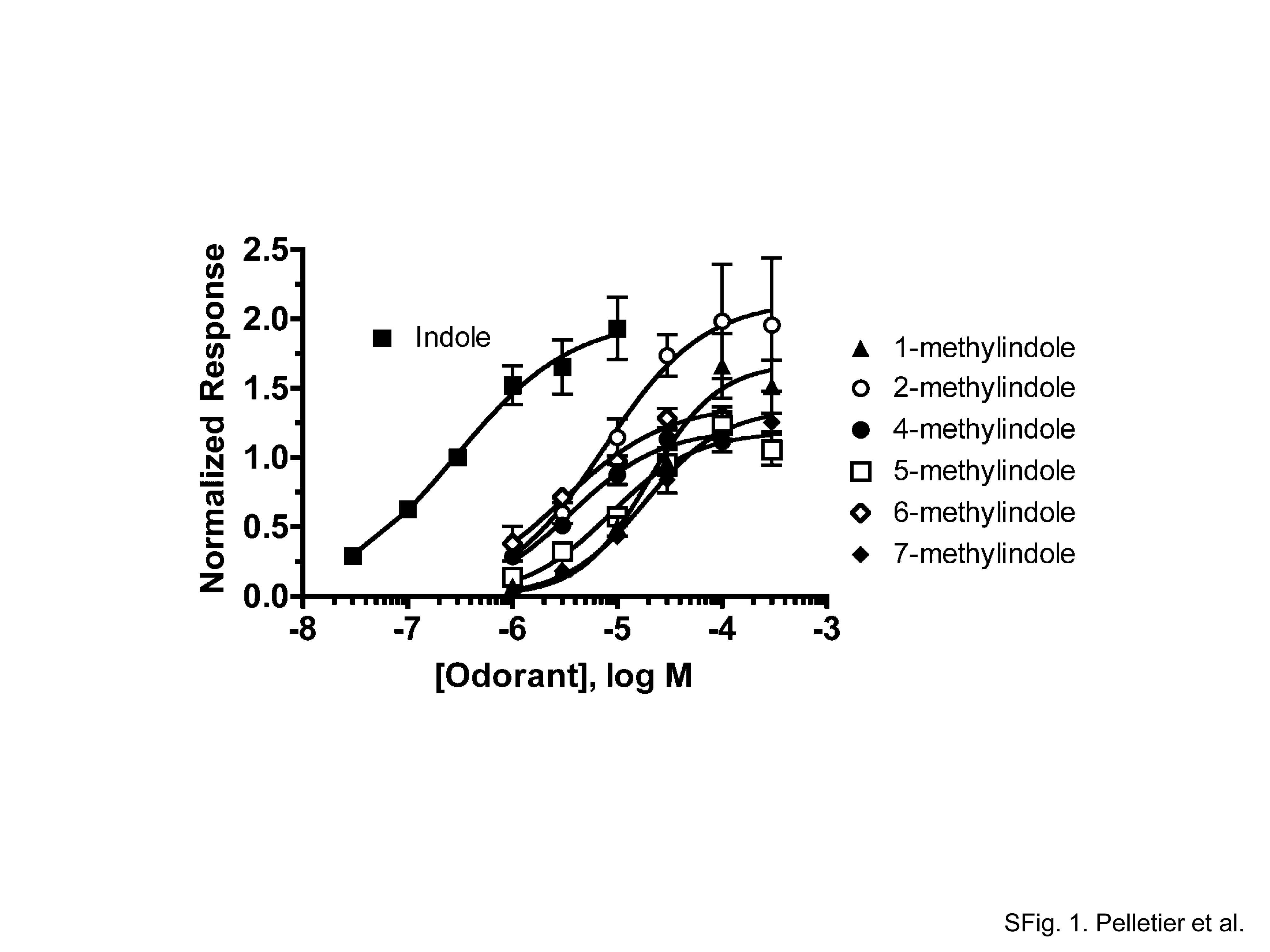

Supplement: Figure S1 — Concentration-response analysis for methylindoles. The concentration-response relationships for CquiOR2 + CquiOR7 expressing oocytes when activated with a range of methylindole concentrations are shown. All data are normalized to the response of each oocyte to 300 nM indole and the curves were fit as described in Materials and Methods (means ± sem; n = 6–9). The data for indole is from Figure 5 and is shown for comparison. EC50 and nH values are provided in Table 1. (1.60 MB TIF) [file pone.0010090.s001.tif]

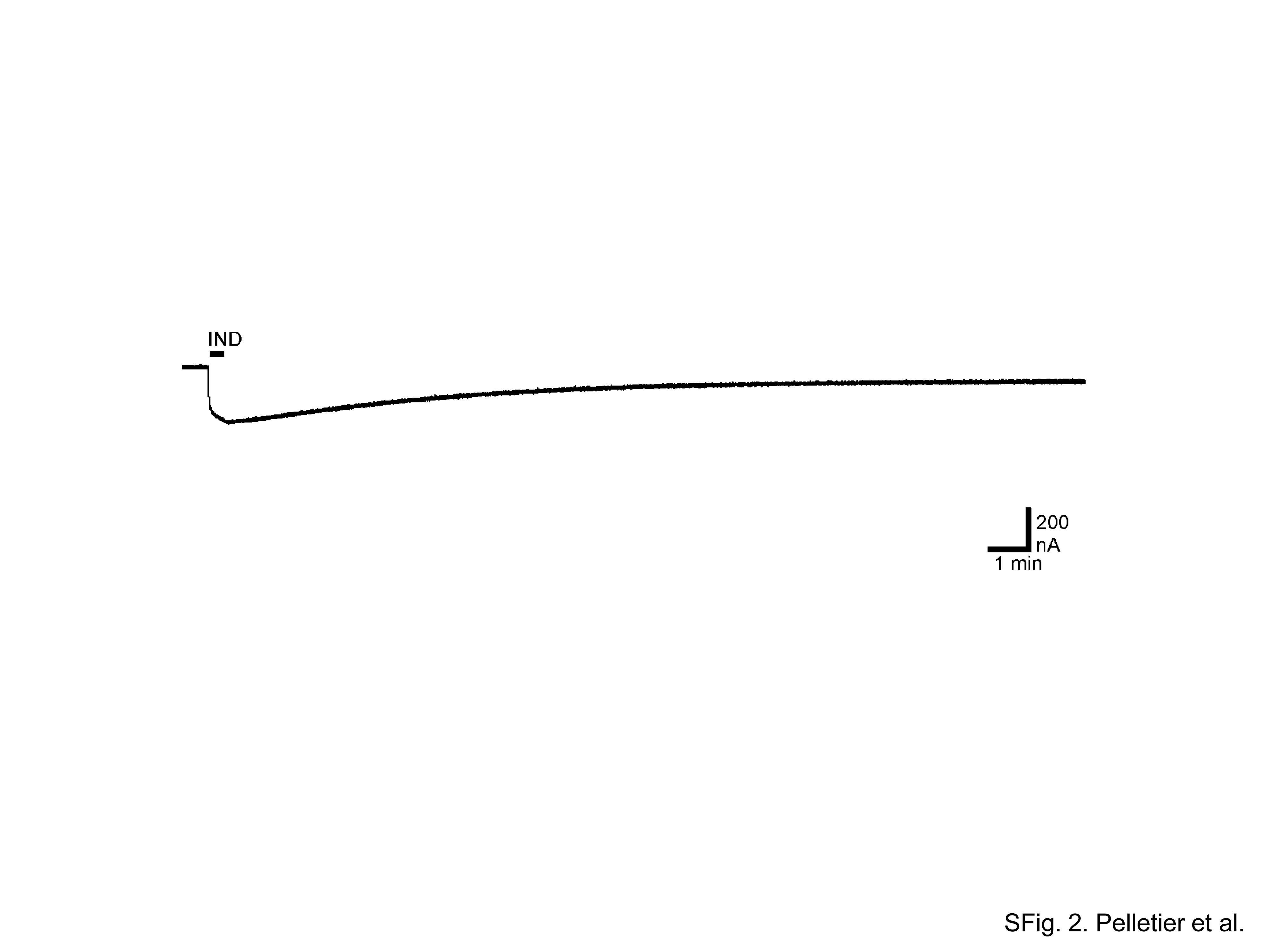

Supplement: Figure S2 — Kinetics of the response of CquiOR2 + CquiOR7 to 10 µM indole. An oocyte expressing CquiOR2 + CquiOR7 is challenged with a 20 s application of 10 µM indole (IND). Note that the response to indole approaches a plateau during the 20 second application. The response diminishes very slowly during washout, suggesting that the receptor is supersaturated and that indole is likely to be highly potent. This is borne out by the concentration-response data in Figure 5. (1.07 MB TIF) [file pone.0010090.s002.tif]
