# Supplementary material for: An Odorant Receptor from the Southern House Mosquito Culex pipiens quinquefasciatus Sensitive to Oviposition Attractants
Source: PLoS One. 2010 Apr 8;5(4):e10090. doi: 10.1371/journal.pone.0010090 (PMC2851645; doi:10.1371/journal.pone.0010090)
Supplement: Table S1 — (0.22 MB DOC) [file pone.0010090.s003.doc]

**Supplementary Information**

**An Odorant Receptor from the Southern House Mosquito *Culex pipiens quinquefasciatus* Sensitive to Oviposition Attractants**

**Table S1. List of 158 putative ORs identified in *Cx. quinquefasciatus***. Putative ORs corresponding to protein accession numbers indicated with an asterisk were not included for phylogenetic analysis as they might represent incomplete and/or wrongly annotated sequences. Full length sequences of CquiOR2 and CquiOR10 were confirmed by cDNA cloning.

| NCBI accession # | VectorBase gene annotation | Size (aa) | NCBI CDD | Putative orthologs (amino acid identity %) |
| --- | --- | --- | --- | --- |
| XP_001842211 | conserved hyp prot | 390 | pfam02949 |  |
| XP_001842212 | conserved hyp prot | 390 | pfam02949 |  |
| XP_001842213* | conserved hyp prot | 314 | - |  |
| XP_001842214 | conserved hyp prot | 391 | pfam02949 |  |
| XP_001842215 | conserved hyp prot | 393 | pfam02949 |  |
| XP_001842216* | conserved hyp prot | 287 | pfam02949 |  |
| XP_001842415 | Odorant receptor 7a | 434 | pfam02949 |  |
| XP_001842654 | Odorant receptor Or1 | 483 | pfam02949 |  |
| XP_001842656 | Odorant receptor Or1 | 427 | pfam02949 |  |
| XP_001842837* | Odorant receptor 7a | 379 | - |  |
| XP_001842892 | Odorant receptor 71a | 364 | pfam02949 |  |
| XP_001842893 | Odorant receptor 94a | 384 | pfam08395  pfam02949 |  |
| XP_001842894 | Odorant receptor 94a | 356 | pfam02949 |  |
| XP_001842895 | Odorant receptor 94a | 379 | pfam02949 |  |
| XP_001843102 | conserved hyp prot | 389 | pfam02949 |  |
| XP_001843498 | conserved hyp prot | 450 | - |  |
| XP_001843880 | Odorant receptor 94b | 375 | pfam02949 |  |
| XP_001843881* | Odorant receptor 94b | 376 | pfam02949 |  |
| XP_001843882* | Odorant receptor 94b | 401 | - |  |
| **XP_001844088**  **CquiOR10** | Odorant receptor Or2 | 376 | pfam02949 | AgamOR10 (70%) AaegOR10 (72%) |
| XP_001844281 | Odorant receptor 83c | 385 | pfam02949 |  |
| XP_001844282 | Odorant receptor 83c | 372 | pfam02949 |  |
| XP_001844283 | Odorant receptor 83c | 381 | pfam02949 |  |
| XP_001845854 | Odorant receptor 13a | 397 | pfam08395  pfam02949 |  |
| XP_001845855 | Olfactory receptor | 486 | pfam02949 |  |
| XP_001845856* | Olfactory receptor | 147 | - |  |
| XP_001845866 | conserved hyp prot | 396 | pfam02949 |  |
| XP_001845876 | Odorant receptor 13a | 389 | pfam02949 |  |
| XP_001845880 | Olfactory receptor | 397 | pfam02949 |  |
| XP_001845884* | Odorant receptor 13a | 479 | pfam02949 |  |
| XP_001845885 | Odorant receptor 13a | 410 | pfam02949 |  |
| XP_001845886 | Olfactory receptor | 421 | pfam02949 |  |
| XP_001845887 | Olfactory receptor | 385 | pfam02949 |  |
| XP_001845888 | Odorant receptor 13a | 401 | - |  |
| XP_001845889 | Odorant receptor 13a | 397 | pfam02949 |  |
| XP_001845890 | Odorant receptor 92a | 396 | pfam02949 |  |
| XP_001845891* | Odorant receptor 13a | 329 | pfam02949 |  |
| XP_001846628 | Odorant receptor 83c | 380 | pfam02949 |  |
| XP_001846629 | Odorant receptor 83c | 394 | pfam02949 |  |
| XP_001846645 | Odorant receptor 7a | 423 | pfam02949 |  |
| XP_001846832* | Odorant receptor 83c | 295 | pfam02949 |  |
| XP_001846834* | Odorant receptor 94b | 310 | - |  |
| XP_001846835 | Odorant receptor 94b | 395 | pfam02949 |  |
| XP_001846836 | conserved hyp prot | 384 | pfam02949 |  |
| XP_001846837 | conserved hyp prot | 333 | pfam02949 |  |
| XP_001846839* | conserved hyp prot | 264 | pfam02949 |  |
| XP_001846840* | conserved hyp prot | 179 | pfam02949 |  |
| XP_001846915* | Odorant receptor 83c | 282 | pfam02949 |  |
| XP_001846919* | Odorant receptor 83c | 309 | pfam02949 |  |
| XP_001846920 | Odorant receptor 83c | 392 | pfam02949 |  |
| XP_001846921* | Odorant receptor 83c | 334 | - |  |
| XP_001846925* | conserved hyp prot | 279 | pfam02949 |  |
| XP_001847323 | conserved hyp prot | 398 | pfam02949 |  |
| XP_001847757* | Odorant receptor 7a | 80 | - |  |
| XP_001847770* | Odorant receptor 83c | 253 | pfam02949 |  |
| XP_001847771* | Olfactory receptor GPRor70 | 253 | pfam02949 |  |
| XP_001847772* | conserved hyp prot | 253 | - |  |
| XP_001847773* | conserved hyp prot | 334 | - |  |
| XP_001847951 | Odorant receptor 94b | 385 | pfam02949 |  |
| XP_001847952* | conserved hyp prot | 368 | pfam02949 |  |
| XP_001847967 | Odorant receptor | 387 | pfam02949 |  |
| XP_001848038* | Odorant receptor 83c | 336 | pfam02949 |  |
| XP_001848424 | Odorant receptor 7a | 436 | - |  |
| XP_001848756 | conserved hyp prot | 370 | pfam02949 |  |
| XP_001848887 | Odorant receptor 83c | 386 | pfam02949 |  |
| XP_001849192* | conserved hyp prot | 298 | - |  |
| XP_001849207 | Odorant receptor 83c | 385 | pfam02949 |  |
| XP_001849428 | Odorant receptor | 387 | pfam02949 |  |
| XP_001849585* | conserved hyp prot | 289 | pfam08395 |  |
| XP_001850048 | Odorant receptor 9a | 385 | pfam02949 |  |
| XP_001850049* | Odorant receptor 9a | 245 | - |  |
| XP_001850050 | Odorant receptor 9a | 396 | pfam02949 |  |
| XP_001850051* | Odorant receptor 9a | 376 | pfam02949 |  |
| XP_001850052 | Olfactory receptor | 412 | pfam02949 |  |
| XP_001850053* | conserved hyp prot | 308 | pfam02949 |  |
| XP_001850054* | Olfactory receptor | 296 | pfam02949 |  |
| XP_001850091 | Odorant receptor 92a | 420 | pfam02949 |  |
| XP_001850369 | conserved hyp prot | 394 | pfam02949 |  |
| XP_001850370 | conserved hyp prot | 394 | pfam02949 |  |
| XP_001850433* | conserved hyp prot | 299 | pfam02949 |  |
| XP_001850434 | conserved hyp prot | 396 | pfam02949 |  |
| XP_001850918 | Odorant receptor 7a | 432 | pfam02949 |  |
| XP_001850951 | conserved hyp prot | 389 | - |  |
| XP_001850952* | conserved hyp prot | 353 | pfam02949 |  |
| XP_001850954* | conserved hyp prot | 305 | - |  |
| XP_001850955 | conserved hyp prot | 411 | pfam02949 |  |
| XP_001850956 | conserved hyp prot | 391 | - |  |
| XP_001851206 | Olfactory receptor | 437 | pfam08395  pfam02949 |  |
| XP_001854083 | conserved hyp prot | 410 | pfam02949 |  |
| XP_001861406 | Olfactory receptor | 383 | pfam02949 |  |
| XP_001861407 | Olfactory receptor | 387 | pfam08395  pfam02949 |  |
| XP_001861602* | Odorant receptor 7a | 238 | pfam02949 |  |
| XP_001862116 | conserved hyp prot | 432 | pfam02949 |  |
| XP_001862530 | Olfactory receptor | 389 | pfam02949 |  |
| XP_001862846* | conserved hyp prot | 238 | pfam02949 |  |
| XP_001862855* | Odorant receptor 7a | 346 | pfam02949 |  |
| XP_001863015* | Odorant receptor 94b | 288 | pfam02949 |  |
| XP_001863016 | Odorant receptor 94b | 340 | pfam02949 |  |
| XP_001863017* | Odorant receptor 94b | 212 | - |  |
| XP_001863018 | Odorant receptor 94b | 401 | pfam02949 |  |
| XP_001863718 | conserved hyp prot | 478 | pfam02949 |  |
| XP_001863842* | conserved hyp prot | 143 | - |  |
| XP_001863915 | conserved hyp prot | 390 | pfam02949 |  |
| XP_001864496 | Odorant receptor | 400 | pfam02949 |  |
| XP_001864497 | Odorant receptor 85d | 394 | pfam08395  pfam02949 |  |
| XP_001864498 | Odorant receptor | 361 | pfam08395  pfam02949 |  |
| XP_001864504 | Odorant receptor 85d | 361 | pfam08395 |  |
| XP_001864506 | Odorant receptor | 396 | pfam02949 |  |
| XP_001864529* | Olfactory receptor | 374 | pfam02949 |  |
| XP_001864542* | Odorant receptor | 368 | pfam02949 |  |
| XP_001864543 | Odorant receptor | 377 | pfam02949 | AgamOR10 (65%) AaegOR9 (65%) |
| **XP_001864544**  **CquiOR2** | Olfactory receptor | 375 | pfam02949 | AgamOR2 (70%) AaegOR2 (81%) |
| XP_001865178 | Odorant receptor 7a | 432 | pfam02949 |  |
| XP_001865179 | Odorant receptor 7a | 433 | pfam02949 |  |
| XP_001865647 | Odorant receptor 56a | 414 | pfam02949 |  |
| XP_001865697 | conserved hyp prot | 416 | - |  |
| XP_001865698* | conserved hyp prot | 279 | - |  |
| XP_001866061* | Odorant receptor 85c | 213 | pfam02949 |  |
| XP_001866094 | Odorant receptor 83c | 400 | - |  |
| XP_001866095 | Odorant receptor 83c | 390 | - |  |
| XP_001866105* | Odorant receptor 7a | 156 | pfam02949 |  |
| XP_001866364 | conserved hyp prot | 406 | - |  |
| XP_001866562 | conserved hyp prot | 390 | pfam02949 |  |
| XP_001866571* | conserved hyp prot | 464 | - |  |
| XP_001866948 | conserved hyp prot | 436 | - |  |
| XP_001867009* | Olfactory receptor | 80 | - |  |
| XP_001867156 | Odorant receptor 7a | 408 | - |  |
| XP_001867186 | Odorant receptor 7a | 416 | pfam02949 |  |
| XP_001867283* | Odorant receptor 7a | 389 | pfam02949 |  |
| XP_001867284 | Odorant receptor 7a | 418 | pfam02949 |  |
| XP_001867285 | Odorant receptor 7a | 418 | pfam02949 |  |
| XP_001867286 | conserved hyp prot | 390 | - |  |
| XP_001867376 | conserved hyp prot | 438 | pfam02949 |  |
| XP_001867492 | Odorant receptor 7a | 354 | pfam02949 |  |
| XP_001867509 | Odorant receptor 7a | 431 | pfam02949 |  |
| XP_001867510 | Odorant receptor 7a | 410 | pfam02949 |  |
| XP_001867512 | Odorant receptor 7a | 414 | - |  |
| XP_001868290* | conserved hyp prot | 357 | - |  |
| XP_001868313 | conserved hyp prot | 396 | pfam02949 |  |
| XP_001868365* | conserved hyp prot | 272 | pfam02949 |  |
| XP_001868367 | Odorant receptor 83c | 396 | pfam02949 |  |
| XP_001868368* | Odorant receptor 83c | 300 | pfam02949 |  |
| XP_001868672 | Odorant receptor 83c | 391 | pfam02949 |  |
| XP_001868673 | Odorant receptor 83c | 393 | pfam02949 |  |
| XP_001868904* | Odorant receptor 7a | 320 | pfam02949 |  |
| XP_001869385 | Odorant receptor | 386 | pfam02949 |  |
| XP_001869386 | Odorant receptor | 383 | pfam02949 |  |
| XP_001869615* | conserved hyp prot | 372 | pfam02949 |  |
| XP_001869676 | Odorant receptor 7a | 435 | - |  |
| XP_001869677 | Odorant receptor 7a | 431 | - |  |
| XP_001869818* | Odorant receptor | 264 | - |  |
| XP_001870055 | Odorant receptor 7a | 410 | pfam02949 |  |
| XP_001870167* | conserved hyp prot | 298 | pfam02949 |  |
| XP_001870184* | conserved hyp prot | 321 | - |  |
| XP_001870516 | Odorant receptor 7a | 431 | pfam02949 |  |
| XP_001870597 | Odorant receptor 83c | 393 | pfam02949 |  |
| XP_001870628* | conserved hyp prot | 300 | - |  |
| **ABB29301** **CquiOR7** | Odorant receptor Or7 | 480 | pfam08395  pfam02949 | AgamOR7 (87%) AaegOR7 (90%) |
